# Valency of Ligand–Receptor Binding from Pair Potentials

**DOI:** 10.1021/acs.jctc.4c00112

**Published:** 2024-03-22

**Authors:** William Morton, Robert Vácha, Stefano Angioletti-Uberti

**Affiliations:** † Department of Materials, Imperial College, London SW7 2AZ, U.K.; ‡ CEITEC—Central European Institute of Technology, Masaryk University, Brno 62500, Czech Republic; § National Centre for Biomolecular Research, Faculty of Science, Masaryk University, Brno 62500, Czech Republic; ∥ Department of Condensed Matter Physics, Faculty of Science, Masaryk University, Brno 62500, Czech Republic; ⊥ Department of Materials, Imperial College, London SW7 2AZ, U.K.

## Abstract

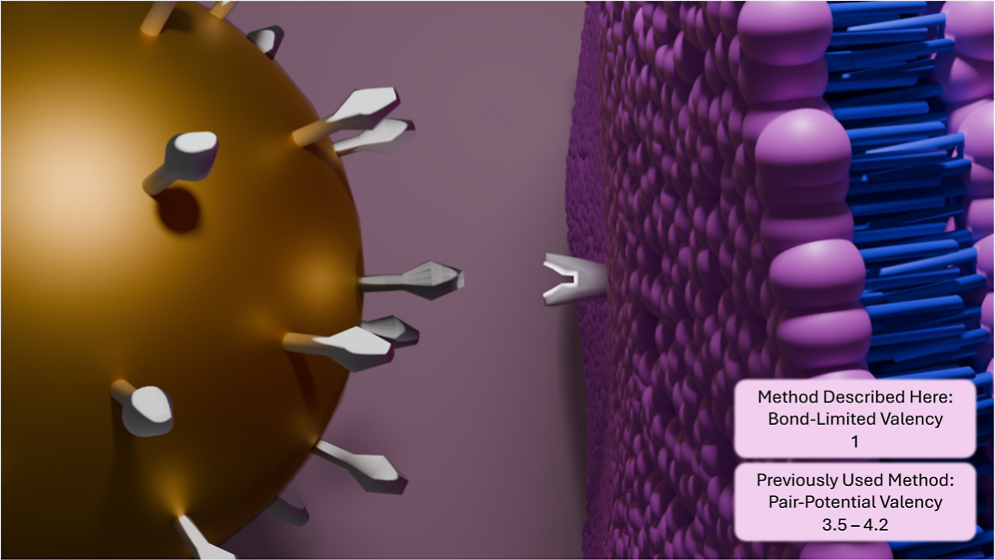

Coarse grained molecular dynamics simulations have been crucial for investigating the dynamics of nanoparticle uptake by cell membranes via ligand–receptor interactions. These models have enabled researchers to evaluate the effects of nanoparticle size, shape, and ligand distribution on cellular uptake. However, when pair potentials are used to represent ligand–receptor interactions, the number of receptors interacting with one ligand, valency, may vary. We demonstrate that the curvature of a nanoparticle, strength of ligand–receptor interactions, and ligand or receptor concentration change the valency, ranging from 3.4 to 5.1 in this study. Such a change in valency can create inaccurate comparisons between nanoparticles or even result in the uptake of smaller nanoparticles than would be expected. To rectify this inconsistency, we propose the adoption of a model based on bond formation and use it to determine the extent to which previous studies may have been affected. This work recommends avoiding pair potentials for modeling ligand–receptor interactions to ensure methodological consistency in nanoparticle studies.

## Introduction

Over the past three decades, the use of nanoparticles as drug carriers has gained considerable attention.^1,2^ The synthesis of various nanoparticle shapes has been leveraged to create nanoparticles with multiple functions, allowing them to be utilized for drug delivery, cell imaging, and photothermal therapies.^3,4^ However, targeted transport and cellular uptake still present several scientific challenges. As an example, nanorods with an aspect ratio of 11.5 were uptaken in HeLa cells to a greater extent than those with smaller aspect ratios, but the mechanisms behind this phenomenon are not fully understood.^5,6^

Ligand–receptor valency is an essential concept in cancer research, where the population of ligands on nanoparticles could be optimized to target specific receptors on a cancer cell surface.^7^ Such optimization relies heavily on a detailed understanding of the ligand’s valency.^8^ For instance, transferrin receptor 1 has been used as a ligand on the surface of nanoparticles to target cancerous cells selectively.^9,10^ Transferrin 1 is an ideal candidate because it is a signaling agent for endocytosis, often overexpressed on cancer cells, and its valency is known to be two, i.e., two ligands can bind to one receptor.^11−13^

Lipid vesicles can be employed as simplified yet representative models of the cell membrane due to their higher reproducibility and improved compositional control.^14^ Here, the uptake of nanoparticles is a spontaneous process—independent of cell signaling pathways, unlike, for example, micropinocytosis.^15−18^ During the wrapping process, the lipid bilayer deforms around the nanoparticle surface, resulting in an increase in bending energy. The Canham–Helfrich Hamiltonian describes the bending energy per unit surface area, as seen in eq 1.^19^
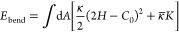
1

In this equation, *H* and *K* are the mean and Gaussian curvature of the surface, κ and κ̅ are the bending and saddle-splay moduli, respectively, and *C*_0_ is the spontaneous curvature. The increased energy that comes with wrapping (eq 1) is balanced by the formation of ligand–receptor bonds.^20^ As long as the energy released from the bonds is greater than the energy needed to bend the membrane, the wrapping will continue.

The bending energy required to wrap a nanosphere is higher for those with smaller radii, which have larger curvatures. With nonspherical nanoparticles, such as nanocubes, nanorods, or nanostars, this relationship can be more difficult to interpret.^21^ For example, it might be energetically favorable for the particle to change orientation while wrapping, as seen with nanorods. Therefore, analyzing how the lipid membrane wraps around the nanoparticle is essential to understanding how cells will interact with it. Unfortunately, imaging of this process requires cryogenic electron microscopy. So, trial and error are needed to find the right concentration and incubation time before freezing to properly capture nanoparticle entry—an expensive and difficult process.^22,23^

Molecular dynamics (MD) simulations of lipid membrane systems offer details about membrane wrapping, capturing the mechanisms and kinetics of nanoparticle uptake. In MD, the uptake dependence on nanoparticle shape, size, and ligand distribution can be analyzed.^24−28^ A standard computational model for these studies relies on ligand–receptor interactions but with no specific ligand or receptor considered. Instead, the model is based on the general concept of short-range adhesion that induces membrane wrapping. While ligands can also be used to translocate nanoparticles through the membrane, here we focus on those that reduce the membrane’s energetic barrier for wrapping a nanoparticle. In vivo, these short-range interactions are usually valence-limited, implying that each nanoparticle ligand and membrane receptor have a maximum number of binding partners.^29,30^ This limitation is primarily due to the excluded volume effects, which prevent extra receptors from binding to the same ligand.

However, in many published articles where MD is used to model nanoparticle endocytosis, ligand–receptor interactions are described by using a purely distance-dependent pairwise interaction. These interactions imply that the valence limitation is not explicitly enforced or controlled.^21,25,26,28,31,32^ Additionally, popular MD engines for coarse-grained simulations such as LAMMPS and ESPResSO lack functions to limit the number of interactions in a pair potential.^33,34^ While a ligand with a valency above 1 is not inherently inaccurate, these systems are specific, and the valency should be highly controlled within the experiment. This paper focuses on delineating the nonphysical effects that can arise in this context as well as proposing a simulation strategy to rectify them.

## Methods

### Membrane Parameterization

All simulations were performed using the LAMMPS program.^33^ Due to the substantial size of most nanoparticle structures, a coarse-grained system is necessary to investigate nanoparticle wrapping. For this reason, coarse-grained membranes have maintained their popularity over the years, and we will focus on the widely used Cooke–Deserno 3-bead lipid model. These lipids are made up of three beads: one to represent the lipid head and the other two to represent the lipid tail.^35^ The three beads are typically connected using finite extensible nonlinear elastic (FENE) bonds, as depicted in eq 2, and the angle between all three beads in the lipids is maintained using the harmonic potential seen in eq 3. The FENE potential as used in LAMMPS includes the Lennard-Jones potential for a short-range repulsion.
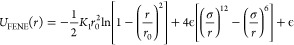
2
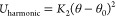
3

In eqs 2–5, *r* is the distance between two beads, ϵ is the Lennard-Jones unit for energy, and σ is the Lennard-Jones unit for distance. The equilibrium bond length between two beads in eq 2, *r*_0_, is 1.5σ, and *K*_1_ is the spring constant with a value of 30ϵ. A harmonic potential is applied to maintain the angle formed by the three beads of each ligand in eq 3, θ_0_, to 180° using a spring constant, *K*_2_, of 10ϵ.

The pairwise potentials between individual beads are described by the Weeks–Chandler–Anderson (WCA) potential in eq 4, and the cosine potential used for lipid tails is shown in eq 5. Equation 4 describes the nonbonding component of the potential energy between individual beads of the lipid model, which in practice is a short-range repulsion that determines the relative size of each bead. Equation 5 facilitates the formation of a lipid bilayer by creating an attractive potential between lipid tails.
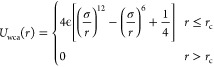
4
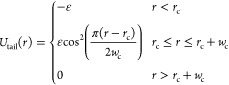
5

The critical length in eq 4, *r*_c_, with *r*_c_ = 2^1/6^*b*, where *b*_head,head_ = *b*_head,tail_ = 0.95σ and *b*_tail,tail_ = σ, is used to maintain a lipid membrane with no intrinsic curvature.^36^ Altering the “*b*” for each bead species is used to determine the relative exclusion radius of each bead. The parameters set here are as they were originally developed by Cooke and Deserno.^37^ As in eq 2, ϵ is the unit of energy used to describe the potential well depth. An additional cutoff parameter (*w*_c_) can be altered with the system’s temperature to change membrane rigidity.

### Simulation Description

The simulated membrane used in this study is composed of 10,452 lipids, 50% of which are receptors that can bind to ligands on the nanoparticle surface. While this results in a larger density of receptors than is biologically relevant, it decreases the simulation time by eliminating diffusion-limited interactions and by relation the nanoparticle wrapping time.^38^ There is no difference between a lipid and a receptor, except for an extra attractive force added between receptors and ligands. The equilibrated membrane dimension is 77 × 77 σ with a WCA cutoff of 1.5σ and *k*_B_*T* = 1ϵ maintained by a Langevin thermostat with a dampening parameter of 1.0τ.^37,39,40^ A larger bilayer is used for nanospheres exceeding 12σ in radius, which is made of 16,296 lipids and is 98 × 98 σ when equilibrated. We simulate a tensionless membrane, where the zero-tension condition is maintained using a Nosé–Hoover barostat in the *XY* plane parallel to the membrane with a pressure dampening term of 1e3τ. Each system was simulated five times for 10^4^τ, with a time-step of 0.01τ.

Ligand–receptor interactions can be modeled using interatomic interaction potentials. A common practice is to use the pair potential in eq 5 that is scaled to be shorter by multiplying the WCA cutoff by 0.3 (WCA_LR_ = 0.45) and making the strength of the interaction ϵ_LR_.^25^ The scaling is intended to help mimic the size and separation of ligand–receptor pairs, and here, unless otherwise stated, ϵ_LR_ = 20ϵ. Using eq 5 is a convenient solution, reducing the number of pair potentials that one must introduce into the system. However, for most MD engines, eq 5 is only available as a pair potential, where valency cannot be imposed. Variations on this potential were also tested for completeness, ensuring that this is not an isolated effect, and can be found in the Supporting Information.

**Figure 1:**
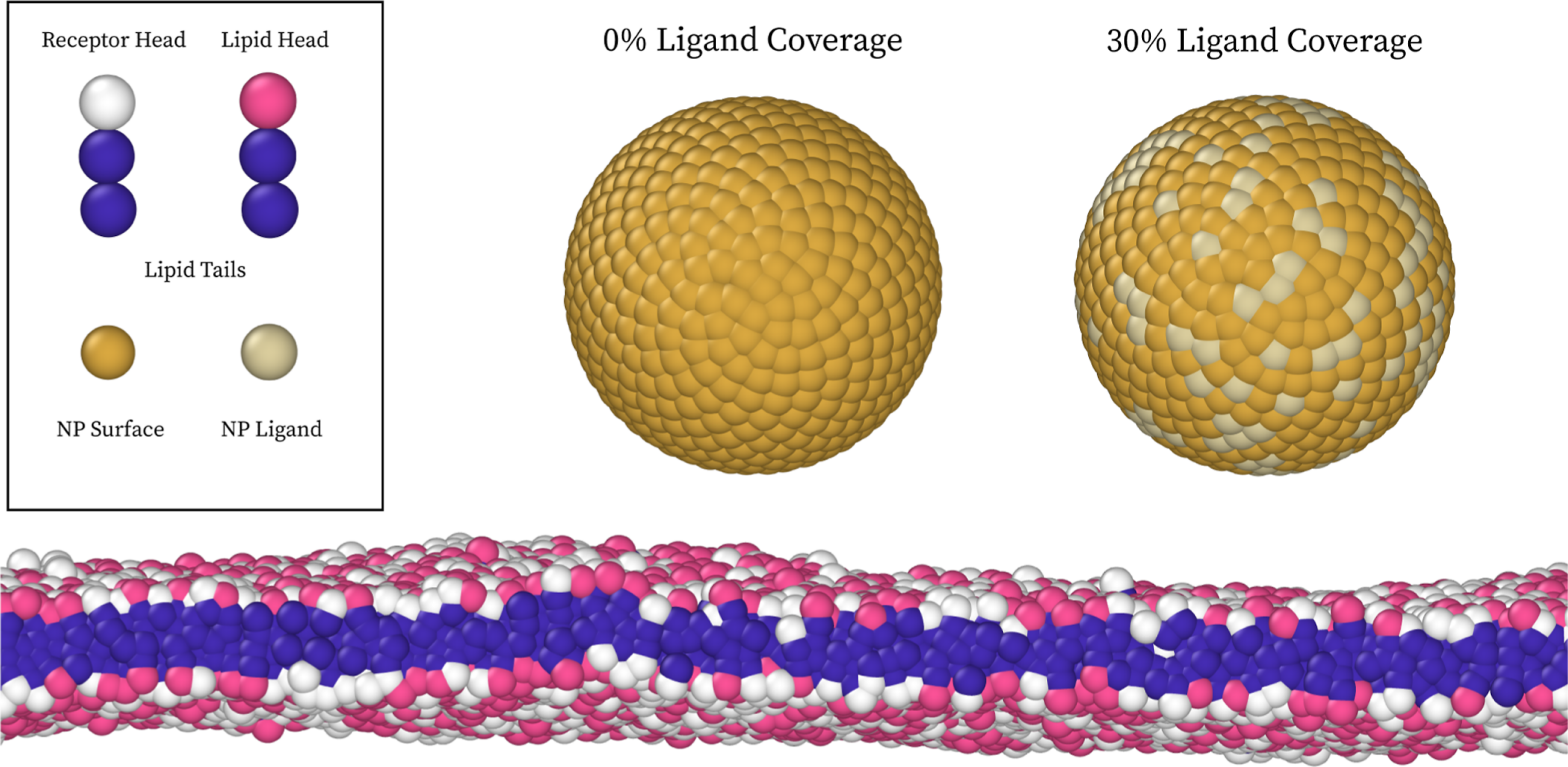
Visualization of the lipid bilayer with two spherical nanoparticles with 0% (left) and 30% (right) of the surface area covered with ligands.

The nanoparticles are generated so that each bead on the surface is roughly 1σ away from its neighbors. Each surface bead utilizes the same WCA repulsive interaction between the head and tail beads of lipids to ensure they do not enter the nanoparticle volume. A representation of the membrane, lipids, receptors, and nanoparticles can be seen in Figure 1.

The thickness of the bilayer membrane can be compared to the experimental results, giving σ. The diffusion of lipids in the membrane can be used similarly to approximate τ. Finally, the elastic properties of the membrane can be used to find ϵ. To do so, we recommend the following methods, which found that membranes similar to ours have σ = 0.9 nm and τ = 1 ns. However, these values will be specific toward the experimental comparison being used. Since we show an effect of the parameterization here, rather than a specific experimental system, we will continue using Lennard-Jones units.

### Imposing and Measuring Valency

In the suggested method where valency is imposed, a tabulated version of the potential in eq 5 is used as a bond style in LAMMPS. Once an unbound ligand and receptor come within 2σ, there is a 50% chance that a bond will be created between the two. The probability of bond formation can be altered to introduce more competition between nearby receptors, a factor that was unnecessary in this study. Bond breaking is allowed within the LAMMPS, but caution must be taken to ensure that the bond energy at rupture is greater than the possible fluctuation in temperature. For this specific system, rupture is set to occur when a bond length exceeds 1.5σ, corresponding to a ligand–receptor binding energy of 1.25ϵ. Since the mean temperature is 1ϵ/*k*_B_ with a standard deviation of 4.4 × 10^–3^ ϵ, there is no concern about a bond length exceeding the dissociation cutoff. At each time step, the conditions were checked for bond formation and breaking. Therefore, since the potential is exactly the same, the only difference between the two methods is that the number of bonds that can form is fixed, maintaining a valency of 1. In this regard, we should highlight that this is not the only method available that can maintain a fixed valency. Sciortino has provided a different algorithm to effectively keep the valence fixed to 1, which introduces a repulsive 3-body potential.^41^ Whereas we only require calculations of 2-body interactions, understanding which algorithm is computationally faster and easier to implement is not straightforward. In fact, it likely depends on the details of the system, the specifics of the MD engine used (e.g., which information is stored at each time step), and the exact implementation of the two algorithms.

In most coarse-grained studies, the absolute value of the wrapping time is inconsequential, and instead, the ratio to that of a sphere is used to gauge the effect of nanoparticle size and shape. These comparisons also help translate the simulated results to the experimental data.

To provide a quantitative definition of valency when using simple distance-dependent interactions, the time average ligand valency is calculated by  after the nanoparticle has been completely wrapped and equilibrated for 100τ. A long equilibration time ensures that the average valency is representative of the final state, where the nanoparticle is in a vesicle.

## Results and Discussion

As described using eq 1, smaller nanoparticles should require more bending energy to wrap. A membrane must induce a large curvature to match the nanoparticle surface. The larger bending energy means that the total ligand receptor binding energy, found by summing the maximum binding energy per ligand on the nanoparticle surface, must increase to counterbalance the bending energy.

We simulated the uptake of a range of spherical nanoparticles with radii ranging from 7 to 12σ. In each simulation performed here, 50% of the nanoparticle surface was covered with ligands having no imposed valency. As larger nanoparticles have more surface area, they would also have a higher number of ligands on the surface, *N*_L_. The interaction strength between ligands and receptors, ϵ_LR_, was scaled so that *E*LR^max^ = *N*_L_ϵ_LR_, would be constant for all fully wrapped nanoparticles regardless of their size. In scaling the interaction, we ensure that the total ligand–receptor binding energy is the same for each nanoparticle if the valency is one, isolating the effect of nanoparticle curvature on valency.

**Figure 2:**
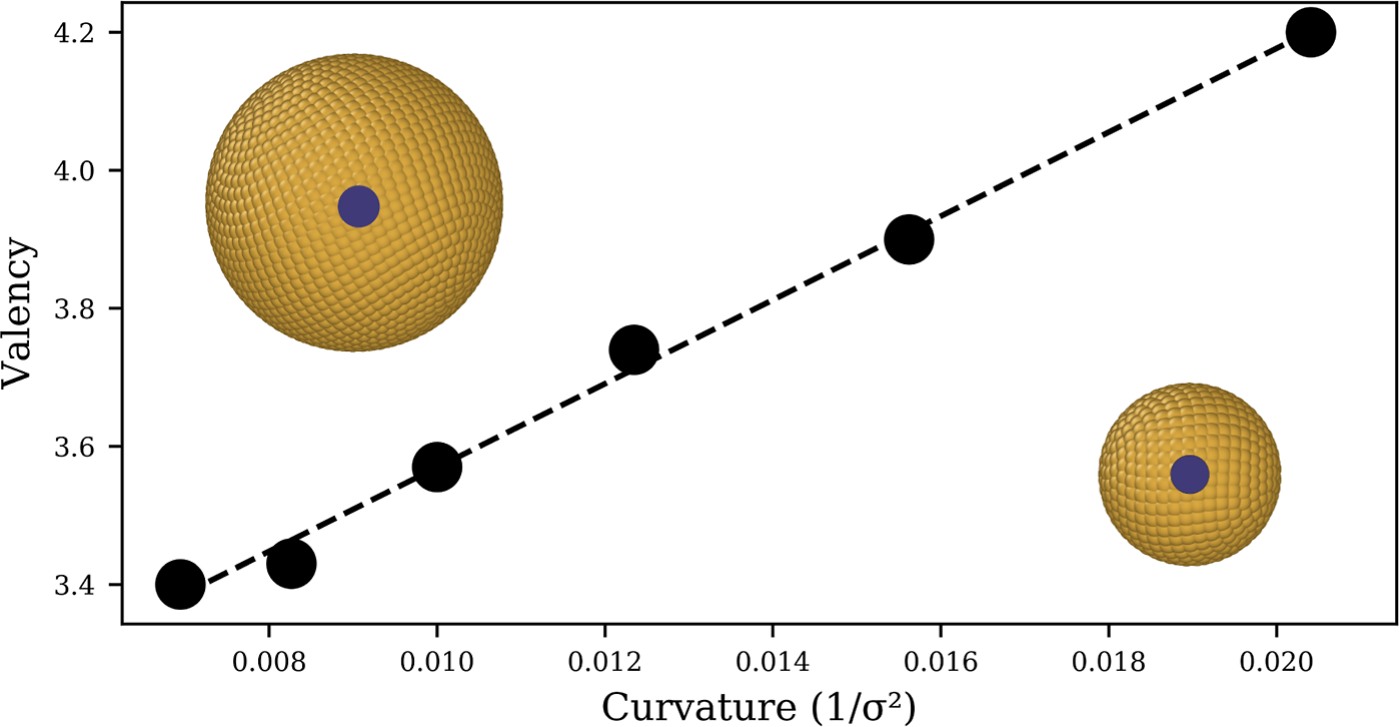
Relationship between the curvature of the nanospheres and the number of bound receptors per ligand (valency). The dotted line is a linear regression of the data points, with a coefficient of determination of 0.99. Nanoparticles of radius 12 (left) and 8 (right) σ are displayed, with the blue circles showing the fraction of the surface that a single lipid can interact with.

Figure 2 shows the linear relationship between the valency and the Gaussian curvature of the nanoparticle. The linearity is likely due to the fractional surface area accessible to receptors in the membrane, which shrinks as a function of *R*^2^. Therefore, a smaller nanoparticle means that a receptor can interact with a larger fraction of the surface. The consequence of the increased valency is that smaller nanoparticles may have an artificially high *E*LR^max^, leading to an incorrect representation of the smallest nanosphere a membrane can endocytose.

**Figure 3:**
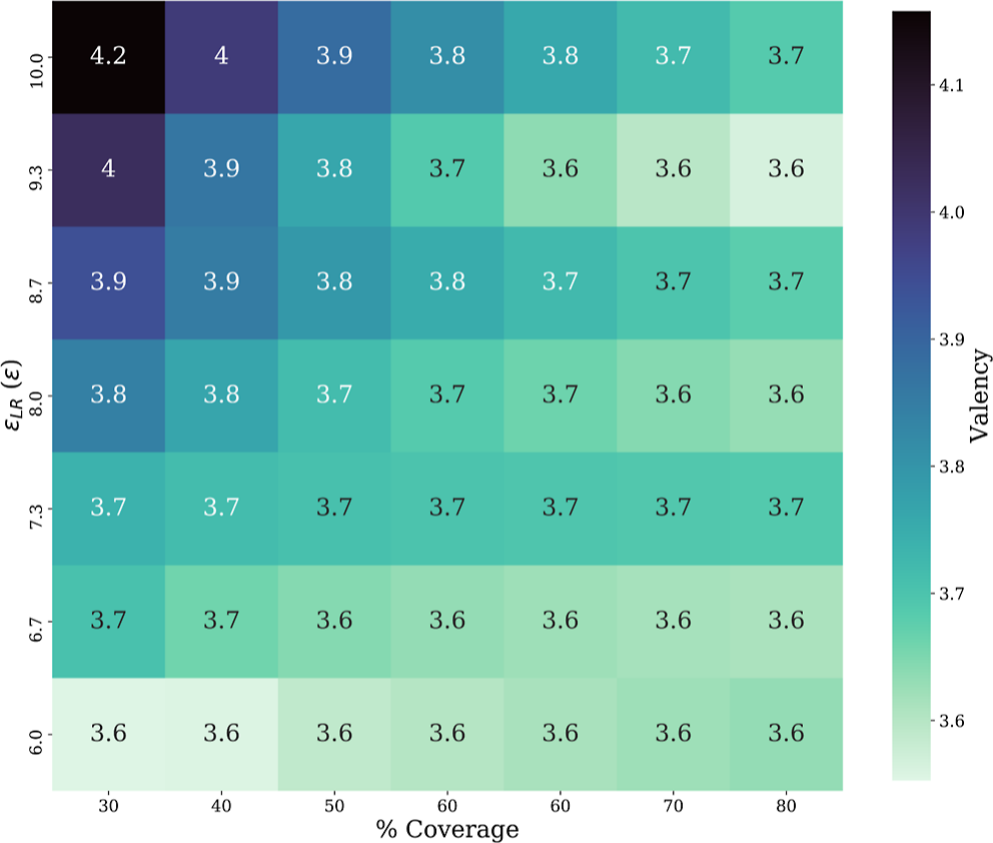
Valency as a function of ligand coverage and ligand–receptor bond energy ϵ_LR_ for a spherical nanoparticle of radius 7σ. Note the monotonic relationship between valency and ϵ_LR_, with a decreasing slope as ligand coverage increases.

Next, the radius of the nanosphere is kept constant at 7σ, while the ligand density and ϵ_LR_ increase. As shown in Figure 3, under these conditions, the valency of ligands changes as a function of both ligand coverage and ϵ_LR_. Initially, the valency increases monotonically with ϵ_LR_ at low ligand coverage. With few ligands present, receptors in the membrane are in competition to bind with ligands on the surface. A higher ϵ_LR_ causes receptors to cluster more densely, with their attractive interaction with ligands counterbalancing the repulsive interaction between receptor heads. But, as ligand coverage is increased, the competitive effect diminishes because there are more ligands to bind with, leading to a saturation effect. While this relationship is expected to hold for each nanoparticle, when valency is not regulated, the exact values are specific to the nanoparticle radius. A larger nanoparticle could accommodate more receptors, and the specific valency may change. This may also be further convoluted by the receptor’s ability to interact with a smaller or larger fraction of the surface area, as mentioned in Figure 2.

Figure 3 suggests that simulation-based studies on nanoparticle endocytosis may contain errors, especially when not constraining valency. These errors are present when comparing effects such as nanoparticle size, ligand coverage, or the strength of ligand–receptor interactions. Although a pair potential could be altered to produce a desired valency under a specific set of conditions, it would need to be tuned for each change in the system.

**Figure 4:**
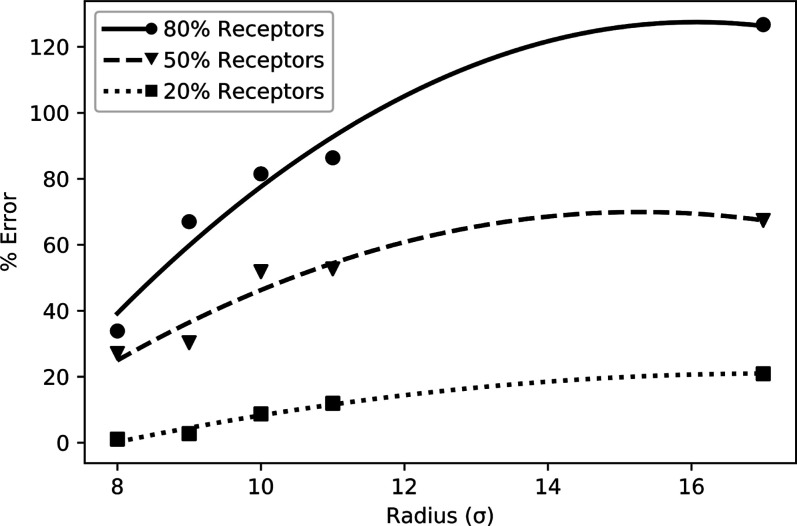
% Error in wrapping time for the valency unlimited method of nanospheres with increasing radii, as described in eq 6. Increasing nanoparticle radius results in a larger valency and higher errors, which are then mitigated by an insufficient number of receptors in the membrane to maintain higher valencies.

Finally, we test how the culmination of these effects may impact a typical study characterizing the uptake speed of a nanoparticle. Two nanospheres’ proportional uptake time should remain consistent across similar models using distance-dependent ligand–receptor interactions. Here, we attempt two different approaches to modeling uptake, with and without imposing a maximum valence. Since valency is the only variable between the two systems, we can determine the scale of errors that can arise from this effect. Three membranes are used, with receptor concentrations of 20, 50, and 80% of the lipid population. As the size of the nanoparticle increases, more ligands will be present on the surface, maintaining 50% coverage. Since we are not isolating the curvature as in Figure 2, each ligand on every nanoparticle will have the same ϵ_LR_. In Figure 4, the percent error of the valence-unlimited system is shown, calculated using eq 6
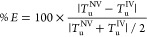
6where *T*_u_^NV^ is the wrapping time of the valence unlimited system and *T*_u_^IV^ is the wrapping time of the system with an imposed valence of *X* = 1.

A parabolic fit is applied to the data in Figure 4 with a coefficient of determination above 0.95 for each receptor concentration as the error is expected to increase with *R*^2^. However, a coarse-grained membrane is not infinite, and the use of an appropriately sized membrane can reduce the need for computational resources. As a result, the membrane may have a limited number of receptors present. For example, the membrane with 20% receptors only has 1045 receptors in total, and the sphere of radius 12σ has 904 ligands. So, there is an insufficient number of receptors present for each ligand to have a valency of two. This suggests that membranes with a lower concentration of receptors restrict the valency in scenarios in which it is not explicitly imposed. As the nanoparticle radius increases, the % error will eventually begin to decrease as receptors become limited.

When the same potential is used as a bond in LAMMPS, instead of a pair potential, it becomes easy to observe the difference in potential energy in the two systems after the nanoparticle has been endocytosed. The difference in potential energy between the two systems, divided by the potential well depth, can provide an approximate scale to the valency of the pair potential being utilized. This assumes that all of the system parameters remain constant. Not every pair potential will be affected equally. However, we have used the most commonly reported one here. Therefore, we recommend that researchers utilize this method as a quick test to identify if their system has any uncontrolled valency.

Due to the artifacts presented thus far, even scaling the effective value of the bond energy ϵ_LR_ to obtain a target valency is not a viable option. The scaling is performed by using the following equation: , where  is the original ligand–receptor bond energy of 20ϵ. Not only does this fail to rectify the original problem, but it also necessitates additional simulations to determine the scaling factors. Table 1 shows the valency for the nanoparticles in Figure 4, along with the valency after ϵ_LR_ has been rescaled.

**Table 1:** Calculated Valency for Nanospheres Interacting with a Membrane Made of 50% Receptors^a^

radius (σ)	ϵ_LR_o__ (ϵ)	*X*_original_	ϵ_LR_ (ϵ)	*X*_scaled_
7	20	5.15	3.87	0.57
8	20	5.07	3.93	0.56
9	20	4.92	4.06	0.58
10	20	4.84	4.12	0.60
11	20	4.74	4.21	0.63
...	...	...	...	...
17	20	4.35	4.59	0.69

a No valency is imposed, and it is shown here that the valency is not easily controlled by scaling ϵ_LR_.

While the error is reduced, there is still no control over the ligand valency.

## Conclusions

Variables that may be considered during an MD study on passive nanoparticle endocytosis, such as the number of ligands on a nanoparticle surface, ligand–receptor binding energy, and size of the nanoparticle, each change the valency of ligand–receptor interactions. Here, valency acts as a confounder, which can misrepresent both the relationship between nanoparticles and lipid membranes and the comparisons between nanoparticles. Attempts to mitigate the inconsistent valency by scaling the interactions between ligands and receptors can improve the accuracy of results but not eliminate the core problem. The study suggests that not all published results will be affected equally. Studies that use lower receptor concentrations reduce the valency when they are not imposed due to the lower probability of multiple receptors diffusing to the same ligand. The trade-off is that lower receptor concentrations result in longer simulation times, which is unnecessary for comparing nanoparticle *T*_u_ if a valence-limited method is used. Those that have scaled interactions may also be able to mitigate the uncontrolled valency to a certain extent. However, the effectiveness of this method is shown here only for spheres. Given the inconsistencies in valency and wrapping time demonstrated throughout this work, the authors recommend that isotropic pairwise functions to model ligand–receptor interactions be discontinued when possible and valency be reported in the methods of each publication.

All the code necessary to recreate the results of this paper and perform simulations in LAMMPS can be found at: https://github.com/shakespearemorton/Nano_Wrap.

These simulations were performed at the Imperial College Research Computing Service (see DOI:10.14469/hpc/2232).

## Supporting Information Available

Form of pair potentials necessary to form a lipid bilayer membrane; experimentally derived interaction energy between transferrin and its receptor; pair-wise interaction between ligands and receptors; total energy in the system from ligand-receptor bonds; modified ligand-receptor pair potentials to examine the effect of shifted and expanded potentials; modified ligand-receptor pair potential built from combining a Lennard-Jones potential with a Gaussian curve; buckled membrane; and valency of modified pair potentials representing ligand-receptor interactions (PDF)
